# Failure of a dietary model to affect markers of inflammation in domestic cats

**DOI:** 10.1186/1746-6148-10-104

**Published:** 2014-05-04

**Authors:** Adronie Verbrugghe, Geert PJ Janssens, Hannelore Van de Velde, Eric Cox, Stefaan De Smet, Bruno Vlaeminck, Myriam Hesta

**Affiliations:** 1 Laboratory of Animal Nutrition, Faculty of Veterinary Medicine, Ghent University, Heidestraat 19, Merelbeke B-9820, Belgium; 2 Department of Clinical Studies, Ontario Veterinary College, University of Guelph, 50 Stone Road East, Guelph, ON N1G 4 V2, Canada; 3 Laboratory of Immunology, Faculty of Veterinary Medicine, Ghent University, Salisburylaan 133, Merelbeke B-9820, Belgium; 4 Laboratory for Animal Nutrition and Animal Product Quality, Department of Animal Production, Faculty of Bioscience Engineering, Ghent University, Proefhoevestraat 10, Melle B-9090, Belgium

**Keywords:** Dietary fat, Feline, Lipid peroxidation, Peroxidisability index, Polyunsaturated fatty acids, Vitamin E status

## Abstract

**Background:**

Oxidative stress and inflammation can be altered by dietary factors in various species. However, little data are available in true carnivorous species such as domestic cats. As numerous anti-inflammatory and anti-oxidative additives become available and might be of use in cats with chronic low-grade inflammatory diseases, the current study aimed to develop a model of diet-induced inflammation by use of two opposite diets. It was hypothesized that a high fat diet enhanced in n-6 PUFA and with lower concentrations of antioxidants would evoke inflammation and oxidative stress in domestic cats.

**Results:**

Sixteen healthy adult cats were allocated to two groups. One group received a moderate fat diet, containing pork lard and salmon oil (AA:(EPA + DHA) ratio 0.19) (MFn-3), while the other group was fed a high fat diet, containing pork lard and chicken fat (AA:(EPA + DHA) ratio 2.06) (HFn-6) for 12 weeks. Prior to and 2, 4, 6, 8, 10 and 12 weeks after starting the testing period, blood samples were collected. Erythrocytic fatty acid profile showed clear alterations in accordance to the dietary fatty acid profile. Serum thiobarbituric acid reactive substances was higher when fed MFn-3 compared to the HFn-6, suggesting augmented oxidative stress. This was associated with a reduced serum vitamin E status, as serum α-tocopherol concentrations were lower with MFn-3, even with higher dietary levels of vitamin E. Serum cytokine and serum amyloid A concentrations were not influenced by diet.

**Conclusion:**

These results point towards a resistance of cats to develop dietary fat-induced inflammation, but also suggest a high susceptibility to oxidative stress when fed a fish oil-supplemented diet even with moderate fat level and additional vitamin E.

## Background

Inflammation as a consequence of multiple acute and chronic disorders in humans and animals is part of the normal innate immune response. However, when inflammation occurs in an uncontrolled manner, it can persist and excessive damage of host tissues and chronic inflammatory diseases such as osteoarthritis, periodontitis, allergic dermatitis and enteritis, asthma, cardiovascular disorders and others, can ensue [1]. Furthermore, it appears that obesity is associated with a low-grade inflammatory process in adipose tissue, resulting in chronic activation of the innate immune system [2]. This can subsequently lead to obesity-associated disorders such as insulin resistance, diabetes mellitus, osteoarthritis, skin problems, lower urinary tract disorders and cardiovascular diseases [3,4]. In addition, oxidative stress commonly defined as an imbalance between oxidants and reductants at the cellular or individual level, results in oxidative damage and plays a role in the initiation and progression of numerous chronic disorders [5,6].

Chronic low-grade inflammation and oxidative stress occur increasingly in humans and companion animals and have become ubiquitous. Furthermore, their effect on health, immunity and general welfare of the individual is deleterious and even life-threatening [7-9].

Research in various species have taught us that specific dietary components, especially fat quantity, fat quality and fatty acid (FA) composition [1,10,11] and antioxidants [11,12], can influence inflammation and oxidative stress. However, only few studies investigated the impact of diet on the inflammatory response in the strict carnivorous cat, consuming a diet with a typically higher fat content compared to omnivores [13].

The present study aimed to test a model of diet-induced inflammation in cats by use of two opposite diets. A theoretical approach was used for diet formulation, based on earlier research in rodents and humans showing that diets with high amounts of fat, altered FA composition, e.g. high in saturated fatty acids (SFA) and n-6 polyunsaturated fatty acids (PUFA) and low in n-3 PUFA, and/or lower concentrations of antioxidants can evoke inflammation and oxidative stress [1,10-12]. It was therefore hypothesised that a diet high in fat, containing higher levels of SFA and n-6 PUFA, but low levels of n-3 PUFA and antioxidants would evoke inflammation and oxidative stress in domestic cats.

## Methods

### Animals and housing

Sixteen domestic short hair cats, six spayed males, two intact males and eight spayed female cats, were included in the study. All cats were adults and aged between 3 and 9 years. Prior to being entered into the study, the cats underwent a physical exam, a jugular blood sample was drawn for complete blood count and serum biochemistry and body weight and body condition score (BCS) [14] were recorded. Cats with a body weight (4.75 ± 0.18 kg) and a normal BCS of 5/9 to 6/9 were included in the study. All cats were healthy and were not given any medication at the time of the study; none had prior medical problems. During the trial cats were kept in their usual group housing.

### Diets and feeding

A theoretical approach to diet formulation was used, creating two opposite diets, based on previous rodent and human studies. Two non-commercial extruded dry cat foods contained the same ingredients yet had different fat content, distinct fat sources, and antioxidant levels in order to create two diets that had in theory opposite inflammatory and oxidative profile. The control diet (MFn-3 diet) was considered to be anti-inflammatory as this diet contained a moderate amount of fat (13.2% crude fat on dry matter basis (DM)); pork lard and fish oil were used as fat sources. Higher levels of antioxidants (125 mg α-tocopherol/kg DM and 1110 μg Se/kg DM) were also present to minimise the peroxidation process and achieve maximum FA preservation. The HFn-6 diet was considered pro-inflammatory as this diet contained 27.6% crude fat on DM, consisting of pork and chicken lard. This diet also contained lower levels of antioxidants (75 mg α-tocopherol/kg DM, 488 μg Se/kg DM), which were still above recommended allowances for adult maintenance according to the National Research Council (NRC), 38 mg/kg DM and 300 μg/kg DM, respectively [15]. The nutrient composition and ingredient list of both diets is shown in Table 1, the FA profile in Table 2. Before and every 4 weeks during the study, the food was analysed for 2-thiobarbituric acid-reactive substances (TBARS) and peroxide value (POV), in order to follow lipid peroxidation over time (Table 3).

**Table 1:** Nutrient composition of two experimental diets with supposed opposite inflammatory and oxidative profile

	**MFn-3 diet**	**HFn-6 diet**
Moisture (%)	10.3	3.7
On DM basis		
Crude protein (%)	31.9	30.0
Crude fat (%)	13.2	27.6
Crude fibre (%)	2.3	1.3
Crude ash (%)	6.6	6.2
NFE^1^ (%)	46.0	34.9
Sugars (%)	1.7	0.4
Starch (%)	36.6	25.7
TDF (%)	14.0	9.8
ME^2^ (kJ/100 g)	1533.0	1927.0
Vit E (mg/kg)	112.0	72.0
Se (μg/kg)	1110.0	488.0
BHA (ppm)	21.0	49.0
BHT (ppm)	138.0	25.0

DM, dry matter; NFE, nitrogen free extract; TDF, total dietary fibre; ME, metabolisable energy; Vit E, vitamin E; Se, selenium; BHA, butylated hydroxyanisole; BHT, butylated hydroxytoluene.

Ingredient list: MFn-3 diet: chicken, corn, rice, corn gluten, lard, beetpulp, herring meal, dried whole eggs, brewer’s yeast, salmon oil, minerals, fructo-oligosaccharides, grape seed extract. HFn-6 diet: chicken, corn, rice, corn gluten, lard, chicken lard, beet pulp, herring meal, dried whole eggs, brewer’s yeast, minerals, fructo-oligosaccharides.

^1^Calculated by subtracting crude protein, crude fat, crude fibre and crude ash from the dry matter content.

^2^Estimated by a 4-step calculation [13].

**Table 2:** Fatty acid profile and peroxidisability index of two experimental diets with supposed opposite inflammatory and oxidative profile

**% of total FA**	**MFn-3 diet**	**HFn-6 diet**
SFA	32.6	35.4
MUFA	36.2	44.3
PUFA	26.3	17.7
C18:2n-6 (LA)	17.95	14.94
C18:3n-6	0.09	0.07
C20:2n-6	0.36	0.36
C20:3n-6	0.11	0.10
C20:4n-6 (AA)	0.53	0.33
C22:4n-6	0.16	0.11
C22:5n-6	0.07	0.01
n-6 PUFA	19.3	15.9
C18:3n-3	1.69	1.48
C20:3n-3	0.12	0.09
C20:4n-3	0.30	0.02
C20:5n-3 (EPA)	1.98	0.06
C22:5n-3 (DPA)	0.82	0.10
C22:6n-3 (DHA)	2.09	0.07
n-3 PUFA	7.0	1.8
n-6:n-3	2.8	8.8
AA:(EPA + DHA)	0.19	2.06
PI^1^	60.9	23.2

FA, fatty acid; SFA, saturated fatty acids; MUFA, monounsaturated fatty acids; PUFA, polyunsaturated fatty acids; LA, linoleic acid; AA, arachidonic acid; EPA, eicosapentaenoic acid; DPA, docosapentaenoic acid; DHA, docosahexaenoic acid; PI, peroxidisability index.

^1^PI, calculated according to Du *et al.* with FA expressed as% of total FA [25].

**Table 3:** 2-thiobarbituric acid-reactive substances and peroxide value of two experimental diets with supposed opposite inflammatory and oxidative profile

		**Week 0**	**Week 4**	**Week 8**	**Week 12**
TBARS (μg malondialdehyde/g food)	MFn-3 diet	1.10	1.43	1.53	1.68
	HFn-6 diet	0.84	0.87	0.77	0.93
POV (meqO_2_/kg fat)	MFn-3 diet	6.37	15.80	17.6	4.53
	HFn-6 diet	3.40	6.55	5.20	2.48

TBARS, 2-thiobarbituric acid-reactive substances; POV, peroxide value.

The amount of food calculated corresponded to the maintenance energy requirement (normal weight cats 418 kJ/kg^0.67^) [16] and was adapted in order to maintain a constant body weight. Cats were fed in group once daily and water was available all day.

### Experimental design

For 4 weeks preceding the trial (adaptation period), all cats were fed the MFn-3 diet as control diet prior to being randomized to one of two groups of eight cats. During the next 12 weeks, one group further continued to receive the MFn-3 diet, while the other group was allocated to the HFn-6 diet. During the study, body weight was recorded weekly and collective food intake daily. Prior to the testing period and 2, 4, 6, 8, 10 and 12 weeks after starting the testing period, fasted blood samples were collected from the jugular vein in both groups. Whole blood was collected in vacutainer tubes containing EDTA for determination of plasma TBARS concentrations and ferric reducing ability of plasma (FRAP) every two weeks and erythrocytic FA profile every four weeks. Plasma was obtained after centrifugation and stored at −20°C until assayed. Erythrocytes were harvested immediately after removing plasma and white blood cells and stored at −20°C until assayed. Serum vacutainer tubes were used to determine serum triglyceride, cholesterol, ascorbic acid, α-tocopherol, Serum amyloid A (SAA) and cytokine concentrations every two weeks. After collection, serum tubes were immediately put on crushed ice protected from light. Within 2 h, serum was obtained by centrifugation and stored at −20°C until assayed. For ascorbic acid analyses, 0.5 mL serum was stabilized by adding 0.75 mL ice cold metaphosphoric acid (3%) and kept at 4°C for 20 min before freezing (−20°C).

The experimental protocol of the present study was approved by the Ethical Committee of the Faculty of Veterinary Medicine, Ghent University, Belgium (EC 2009/151), and was in accordance with institutional and national guidelines for the care and use of animals.

### Analytical methods

Diets were analysed for DM, ash, crude protein (ISO 5983–1, 2005), crude fat (ISO 6492, 1999), crude fibre [17], total dietary fibre (TDF) [18]. Metabolisable energy was estimated using predictive equations according to a four-step calculation based on calculation of gross energy and digestibility of energy [16]. The method as described by Brübacher *et al.* was used for dietary α-tocopherol analysis [19]. Total dietary selenium (Se) content was determined after microwave digestion, by inductively coupled plasma atomic emission spectroscopy (ICP-AES) following hydride generation. Phenolic antioxidants (BHT, BHA) were analysed by gas chromatography with flame ionization detector (GC-FID) following acetonitrile extraction. The POV of the diets was determined by iodometric titration according to Gray [20]. Dietary lipid oxidation was assessed by TBARS measurement using the distillation method described by Tarladgis *et al.* and was expressed as μg malondialdehyde (MDA)/g food [21]. Dietary FA were extracted using chloroform:methanol (2:1, vol/vol), modified based on the methods of Folch et al. [22]. FA in extracted dietary lipids and erythrocytes were methylated and fatty acid methyl esters were extracted. For food samples, hexane extract was directly used for injection. Extracted FA methyl esters from erythrocytes were dissolved in 200 μL of hexane prior to injection. FA methyl esters were determined by gas chromatography as described by Van Ranst et al. [23]. Ratios; n-6:n-3, AA:(EPA + DHA), AA:DHA and AA:EPA were calculated as described by Filburn & Griffin [24]. The peroxidisability index (PI) was calculated using the formula described by Du et al. [25], with the FA concentration expressed as % of total FA.

Plasma TBARS concentrations, reflecting lipid peroxidation, were determined as described by Vossen *et al.*[26]. The plasma FRAP value, a measure of ‘the total antioxidant power’, was determined as described by Vossen *et al.*[26]. Serum triglycerides and cholesterol concentrations were determined enzymatically using a commercially available method (Triglycerides; Cholesterol Randox Laboratories, Crumlin, UK). Serum concentrations of ascorbic acid were analysed by reversed phase HPLC and fluorescence detection following precolumn derivation. Serum α-tocopherol concentrations were determined by reversed phase HPLC and UV detection. The acute phase protein, SAA, was measured according to Hansen *et al.*[27]. Cytokines; TNFα, IL6 and, IL10 were determined using a feline specific commercially available ELISA kit (R&D systems, Inc, Minneapolis, USA). Lower detection limit of these cytokines was respectively 15.6 pg/ml, 31.25 pg/ml, and 125 pg/ml.

### Statistical analyses

Statistical analyses were performed using the Superior Performing Software System (SPSS) version 17 (SPSS Inc., Illinois, USA). As the cats were group-housed feed intake for individual cats could not be measured. However, as all cats maintained their body weight statistical analyses was performed using cat as experimental unit (n = 16) instead of group. Normality of distribution was examined using the Kolmogorov-Smirnov test, prior to further analyses. Normally distributed data were statistically analysed by repeated measures ANOVA with time as within subject factor, diet as between subject factor and measurement at time point 0 (before the testing period) as co-variable. As data of cytokines TNF-α, IL-6 and IL-10 were not normally distributed, a nonparametric analytical method, namely Mann–Whitney, was used to investigate differences between diets at each time point. Statistical significance was accepted at P < 0.05.

## Results

None of the cats refused to eat any of the diets and none showed signs of illness or maldigestion. Mean energy intake was comparable in both groups (MFn-3 diet: 817.4 kJ/d/cat; HFn-6 diet: 871.8 kJ/d/cat). Body weight (time: *P* = 0.494; time × diet; *P* = 0.229; diet: *P* = 0.818) and BCS remained stable in all cats during the study, and were not affected by the test diets.

### FA-profile

The dietary FA profile (% of total FA) as shown in Table 2, corresponded with the fat sources in the diets. Compared to the HFn-6 diet, the MFn-3 diet contained lower concentrations of SFA and mono-unsaturated fatty acids (MUFA), yet the total PUFA content remained the same. The MFn-3 diet also contained higher levels of n-3 PUFA, especially eicosapentaenoic acid (EPA), docosapentaenoic (DPA) and docosahexaenoic acid (DHA), whereas the HFn-6 diet contained higher levels of n-6 PUFA, especially linoleic acid (LA). N-6:n-3 ratio was 2.8 for the MFn-3 diet, compared to 8.8 for the HFn-6 diet. The AA:(EPA + DHA) ratio was 0.19 for the MFn-3 diet, and 2.06 for the HFn-6 diet.

**Figure 1:**
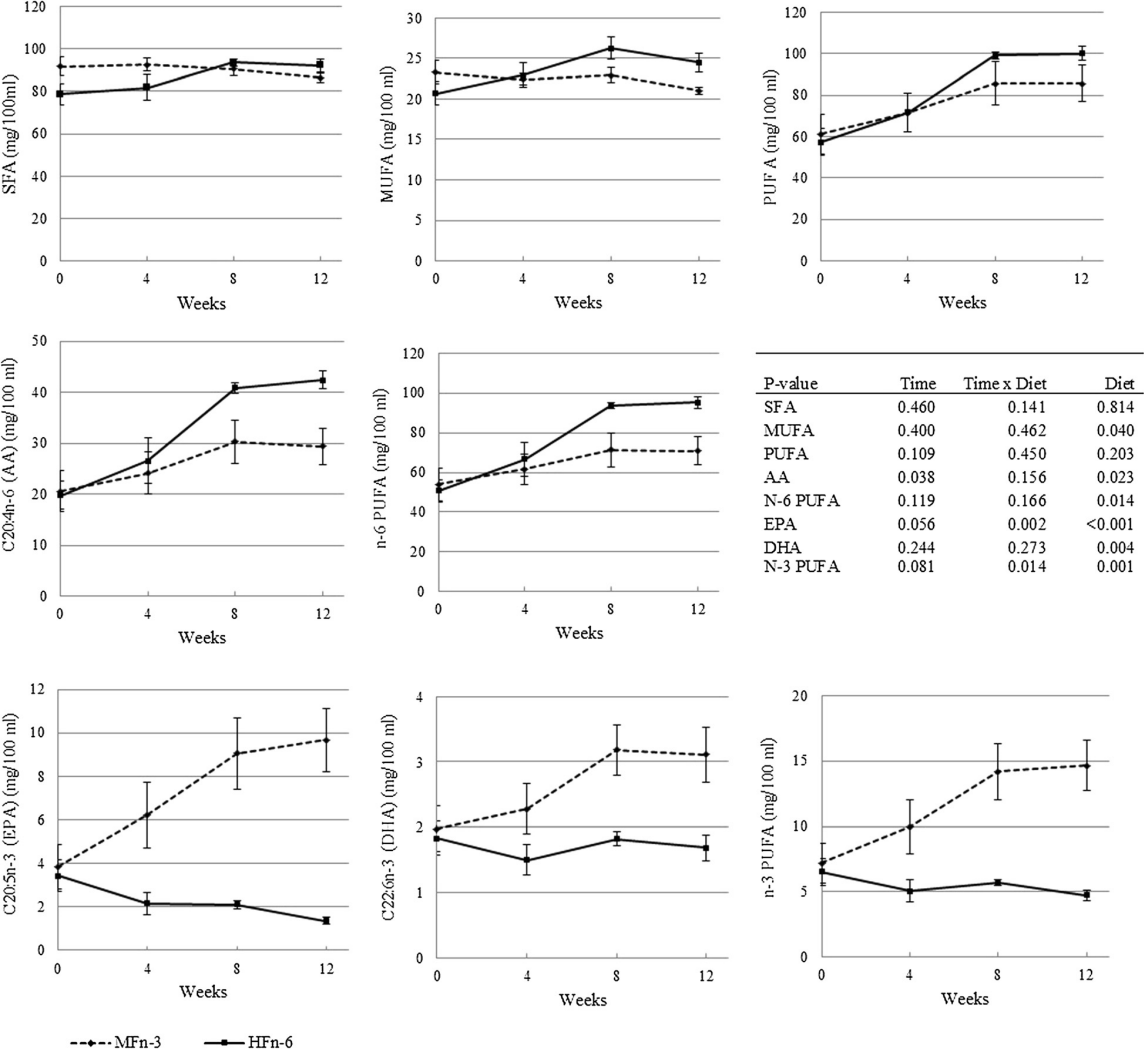
Mean erythrocytic fatty acid profile ± SEM in 16 cats fed one of two non-commercial extruded dry cat foods with supposed opposite inflammatory and oxidative profile, during a 12-week period.

The erythrocytic FA profile (Figure 1) was consistent with the FA profile of the diets. The concentrations of SFA and total PUFA (Figure 1), expressed as mg/100 mL, did not show any differences over time and between diets. No time effect was observed for the MUFA concentrations, yet a diet effect, i.e. higher with the HFn-6 diet, was noted. Concerning n-6 PUFA, total n-6 PUFA, arachidonic acid (AA) and LA (time: *P* = 0.342; time × diet: *P* = 0.195; diet: *P* = 0.016, data not shown) concentrations were significantly higher when fed the HFn-6-diet compared to the MFn-3 diet. A time effect was only noted for AA. With regard to n-3 PUFA, total n-3 PUFA, EPA, DPA (time: *P* = 0.034; time × diet: *P* = 0.156; diet: *P* = 0.032, data not shown) and DHA concentrations were significantly higher in cats fed the MFn-3 diet compared to the HFn-6 diet. A time effect was noted for total n-3 PUFA, EPA and DPA. N-6:n-3 ratio increased when fed the HFn-6 diet from 8.26 ± 0.6 to 20.74 ± 1.2 and decreased when fed the MFn-3 diet from 8.26 ± 0.6 to 5.35 ± 0.6 (time: *P* = 0.254; time × diet: *P <* 0.001; diet: *P <* 0.001, data not shown). Also AA:EPA + DHA (time: *P* = 0.278; time × diet: *P <* 0.001; diet: *P <* 0.001, data not shown), AA:DHA (time: *P* = 0.046; time × diet: *P <* 0.001; diet: *P <* 0.001, data not shown), AA:EPA ratios (time: *P* = 0.066; time × diet: *P* = 0.001; diet: *P* < 0.001, data not shown) increased over time for the HFn-6 diet, compared to the MFn-3 diet.

### Oxidative indices

The calculated PI was higher for the MFn-3 diet compared with the HFn-6 diet (Table 2) and dietary TBARS and POV were higher at each time point for the MFn-3 diet (Table 3). It was also noticed that POV increased in both diets until 8 weeks, but decreased at week 12.

**Figure 2:**
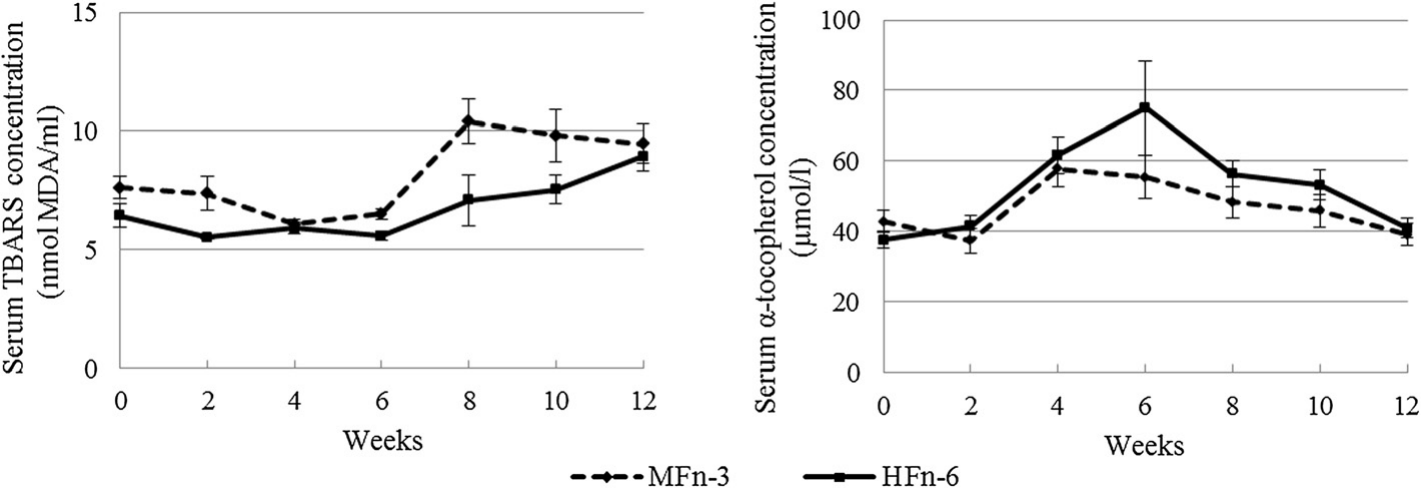
**Mean serum 2-thiobarbituric acid-reactive substances (TBARS) and α-tocopherol ± SEM concentrations in 16 cats fed one of two non-commercial extruded dry cat foods with supposed opposite inflammatory and oxidative profile, during a 12-week period.** P-value: TBARS: time: P = 0.250; time × diet: P = 0.912; diet: P = 0.003, α-tocopherol: time: P = 0.322; time × diet: P = 0.595; diet: P = 0.012.

As shown in Figure 2, serum TBARS concentrations were higher in cats fed the MFn-3 diet compared to the HFn-6 diet. In contrast, serum α-tocopherol concentrations were lower when fed the MFn-3 diet compared to the HFn-6 diet (Figure 2). However, α-tocopherol:triglyceride ratio and α-tocopherol:cholesterol ratio as well as serum triglycerides and cholesterol concentrations showed neither diet nor time effects (data not shown). For serum FRAP and ascorbic acid concentrations no significant time or diet effects were noted (data not shown).

### Inflammatory indices

No differences between diets were observed at any time point for the pro-inflammatory cytokines TNFα and IL6; and anti-inflammatory cytokine IL10, as well as for the TNFα:IL10 ratio (data not shown). SAA concentrations were below the detection limit (1 mg/L) in all cats at all times.

## Discussion

As expected from earlier studies in cats [28,29] and dogs [24], modulating the dietary fat content and fat sources induced pronounced changes in the dietary FA composition, which led to similar changes in the erythrocytic FA profile. In accordance with differences in dietary FA profile, erythrocytic n-6 PUFA and LA concentrations where increased with chicken fat administration. In contrast, erythrocytic n-3 PUFA, EPA, DPA and DHA concentrations were increased with fish oil administration. Whereas no differences in dietary AA concentrations were noted between both diets, lower erythrocytic AA concentrations were observed when fish oil was added to the diet. This observation is due to the incorporation of EPA and DHA in the cell membrane of red blood cells at the expense of AA [1]. In addition this is the first feline study that assessed the erythrocytic FA profile to evaluate FA status and the first to show how much time is necessary for erythrocytic FA stabilisation. At week 8, the erythrocytic FA profile, especially the n-6:n-3 ratio, seemed to stabilise in cats receiving the MFn-3 diet, while the n-6:n-3 ratio was still increasing from week 8 to week 12 in cats receiving the HFn-6 diet. It is important to note that the MFn-3 diet was also fed as a control diet to all cats for 4 weeks preceding the study. This way all cats had similar basal values when the testing period was started. Based on a theoretical approach to diet formulation it was expected that feeding the MFn-3 diet would induce an anti-inflammatory state prior to induction of inflammation with the HFn-6 diet. This could explain the earlier occurrence of stabilisation of the erythrocytic FA profile in cats fed the MFn-3 diet and means that about 12 weeks are necessary to achieve erythrocytic FA stabilisation, which is consistent with an approximate half-live of 50 days for red blood cells.

Whereas it was expected from literature that the HFn-6 diet, high in fat, containing high levels of SFA and n-6 PUFA, but low levels of n-3 PUFA would stimulate inflammation [1,10-12], inflammatory indices remained unaltered by feeding this diet. This absence of effect can be due to several reasons. A first factor may be species differences. Cats utilize fat efficiently and tolerate fat better than most other studied species. According to the NRC, the maximal fat content of balanced diets for cats may be reasonable high without any known adverse effects, apart from total calories [13]. The HFn-6 diet used in the present study contained 65%ME from fat. Whereas, in general feline diets span a wide range from 22 to 55%ME, depending on cat food category [13]. Yet, the dietary fat amount in the present diet did not exceed the recommended safe upper limit, which is approximately 70%ME (33 g/kgDM) [13]. Moreover, the higher amounts of LA in the HFn-6 diet may not have contributed to AA synthesis due to the lack of delta-6 desaturase [30,31]. This may partly explain the lack of an inflammatory response in the cats fed the HFn-6 diet. Secondly, the methods used to evaluate inflammation also have limitations, especially the absence of an inflammatory trigger. SAA, a positive acute phase protein, was below the detection limit in all cats. In contrast, Kajikawa *et al.* showed increased SAA concentrations in hospitalized or diseased cats and in cats with experimentally induced inflammation (LPS or turpentine oil) compared to clinically normal cats [32]. The latter conditions coincided with acute inflammatory responses, while no acute trigger was used in the present trial as chronic low-grade inflammation was of interest. Also serum cytokine (TNFα, IL6, IL10) concentrations remained unchanged throughout the present study. In dogs, decreasing the n-6:n-3 ratio did not alter serum IL1, IL6, TNFα concentrations in the absence of a trigger [33], while serum IL1 and IL6 concentrations increased to a lesser extent after LPS injection [34]*.* Therefore, modulation of the FA profile is probably a faint inflammatory trigger. Increasing the fat content might work as a better trigger. High-fat-diet-induced inflammation is used extensively as model to explore the inflammatory response in rodents [35-37]. Future studies should investigate the impact of dietary lipids on cytokine gene expression profiles and should also assess cytokine profiles following stimulation of mononuclear cell cultures with mitogens. Still, interfering factors may also be a reason, especially as the HFn-6 diet contained a higher amount of MUFA compared to the MFn-3 diet. This difference was also observed in the feline red blood cells as the erythrocytic MUFA concentration was higher when cats were fed the HFn-6 diet. In humans, MUFA-enriched diets are thought to offset pro-inflammatory effects of high fat diets [38-40]. So far, data on the anti-inflammatory effect of MUFA are not available in cats, and as the diets used in this first study differed in many aspects, more research is warranted to investigate the potential inflammatory effects of each dietary lipid component separately to eliminate interfering factors.

Regarding lipid peroxidation, feeding the MFn-3 diet resulted in higher serum TBARS concentrations, reflecting the peroxidation of lipids by free radicals. This observation was in accordance with the higher PI observed for this diet, which was mostly due to the addition of fish oil and its increased vulnerability to oxidative degradation [41,42]. The PI value for the MFn-3 diet doesn’t seem extremely high compared to values from experimental diets used for rodent studies [25]. Still, data on the PI values of cat foods is not available. In addition, dietary TBARS and POV were also higher for the MFn-3 diet at each time point throughout the study. The highest POV reported in this study was 17.6 mEqO_2_/kg fat. In comparison, the POV of fresh oils are reported to be less than 10 mEq/kg fat and a rancid taste often begins to be noticeable to humans when the POV is between 20 and 40 mEq/kg fat [43]. To date, the NRC, the American Association of Feed Control Officials (AAFCO) and the European Pet Food Industry Federation (FEDIAF) have not set recommended limits for POV in pet food. It is also unclear whether diets with mildly elevated POV have adverse health effects in cats. Food refusal has been reported in cats fed oxidised diets, yet some cats continue to eat such diets. A 15% reduction in food consumption was observed in cats fed a diet with a POV of 135 mEqO_2_/kg fat, yet no weight loss occurred [44]. In dogs, consumption of oxidised dietary fat (100 and 200 μg aldehydes/g food) resulted in slower puppy growth, suppressed immunity and reduced serum vitamin E levels [45]. To minimize peroxidation of the added very-long-chain n-3 PUFA, higher levels of α-tocopherol and Se were added to the MFn-3 diet. Nonetheless, higher serum TBARS concentrations were observed with this diet, suggesting oxidative stress. Also the serum vitamin E status was markedly lower when feeding the MFn-3 diet, despite the higher dietary α-tocopherol intake when feeding the MFn-3 diet (6.8 mg/d) compared to the HFn-6 diet (1.8 mg/d), meaning that the amount of α-tocopherol that was present, was not enough to scavenge free radicals and to compensate for the increased peroxidation that was related to the MFn-3 diet. Still, both diets contained an amount of α-tocopherol (MFn-3 diet 125 mg/kg DM; HFn-6 diet 75 mg/kg DM) which was higher than the recommended allowances for adult maintenance (38 mg/kg DM) according to the NRC [46]. For high-PUFA diets, the NRC recommends higher levels of α-tocopherol (120 mg/kg DM) [46]. The MFn-3 diet with added fish oil contained an amount of α-tocopherol which was above this recommendation. In humans, Harris and Embree proposed that a ratio of at least 0.6 mg of tocopherol per gram of PUFA should be maintained in the diet [47]; meaning that both diets used in the present study were also in agreement with this recommendation (MFn-3 diet: 2.6; HFn-6 diet: 1.5). However, not only the PUFA content, which was similar among diets, but also the amount of specific FA, play an important role in the initiation of lipid peroxidation; the greater the degree of unsaturation the more vulnerable to lipid peroxidation [41]. Muggli proposed a formula taking into account mixtures of FA [33]. Both diets used in the present study contained higher levels of α-tocopherol compared to this recommendation (calculated according to Muggli [48]: MFn-3 diet: 41.2 vitamin E mg/kg diet; HFn-6 diet: 42.0 vitamin E mg/kg diet). In addition, the dietary vitamin E concentration necessary to protect against lipid peroxidation also depends on the concentration of Se. Dietary Se concentrations (MFn-3 diet: 1110 μg/kg DM; HFn-6 diet: 488 μg/kg DM) also exceeded the recommended allowance according to the NRC (300 μg/kg DM) [15]. The markedly lower vitamin E status when feeding the MFn-3 shows that vitamin E is a limiting factor in the appearance of lipid peroxidation and that the amounts of α-tocopherol added to the MFn-3 diet were not enough to prevent oxidative stress. Nonetheless, dietary α-tocopherol concentrations were in accordance with recommendations found in literature. Similar findings were observed in dogs fed a high n-3 PUFA diet [45,49]. Still, it is unknown whether this problem is of biological value. Moreover, cats seemed to be able to adapt, as serum α-tocopherol concentrations reached nearly identical values for both diets at 12 weeks. More research is warranted to investigate the biological effect of lipid peroxidation products and the decrease in vitamin E status with high n-3 PUFA diets. Further studies are also required to set species-specific recommended allowances of vitamin E and Se and to investigate other possible factors influencing the appearance of lipid oxidation and oxidative stress.

## Conclusion

Changing dietary fat content, FA profile and anti-oxidant concentration in cats altered the erythrocytic FA profile as expected from the dietary FA composition. The absence of alterations of inflammatory indices suggests resistance of cats to develop dietary fat-induced inflammation. Still, more research is warranted to investigate the potential inflammatory effects of each dietary lipid component separately to eliminate interfering factors using more sensitive methods for the assessment of inflammation. Feeding a moderate fat diet supplemented with very long chain n-3 PUFA augmented oxidative stress and was associated with a reduction of the serum vitamin E status, even with higher dietary levels of vitamin E, suggesting a high susceptibility of cats to oxidative stress and calls for caution when feeding fish oil-supplemented diets to cats. Further studies are required to define the appropriate dietary level of vitamin E and Se when feeding high PUFA diets to adult cats, in order to prevent oxidative stress.

## Competing interests

The authors declare that they have no competing interests.

## Authors’ contributions

AV was responsible for the study design, study performance, laboratory analyses, data analyses and manuscript drafting. MH and GPJJ, both mentors of AV, contributed to the development of the study design and data analyses. HV participated in the study performance and cytokine analyses. EC supervised the cytokine analysis. SD and BV supervised plasma and food analyses of fatty acids and oxidative indices. All authors contributed to data analyses and manuscript drafting.
